# The "Lancet" and the Hyderabad Commissions on Chloroform

**Published:** 1897-06

**Authors:** 


**The "Lancet" and the Hyderabad Commissions on Chloroform.** Pp. viii., 193. London: The Offices of the *Lancet*. [N.D.]

Three investigations had already been made on the action of chloroform—by the Royal Medical and Chirurgical Society, by the Glasgow Commission, and by the first Hyderabad Commission—when, further to test the finding of the last-mentioned, the members of the second Hyderabad Commission were called together in 1889. After a long course of research, involving experiments on 430 animals, the Commissioners stated that they saw no reason to withdraw from their previously published opinion, and that they had arrived at identically similar results from experiments carried out by all methods and under all conditions that human ingenuity could devise.

For the finding of the first Commission we must refer our readers to the book under review.

In the 1889 or second Hyderabad Commission, we venture to say that the work was conducted in a much more scientific and thorough manner. Monkeys, as nearest approaching the human subject, were employed, as well as dogs and other animals, and the "chloroform administered to them in every possible way and under every conceivable condition." Diseased and healthy animals were used, drugs were administered in some cases beforehand, large meals were given before the experiment or the dogs were kept starving, the chloroform was given with inhalers or without, in large or small doses, with the animals in all positions, and with the one invariable result that respiration ceased before the heart's beat. In a tabulated form is given the time elapsing between the cessation of respiration and the heart's action in uncomplicated cases, the observations being made partly by auscultation, partly from the movements of a needle inserted into the heart, and partly by observation after opening the thorax. In connection with this it will be interesting to observe that artificial respiration was found to be nearly always efficacious within 28 seconds of the cessation of natural respiration, but in the 18 experiments in which morphia had been previously injected this time of possible recovery was materially shortened. This fact is of much importance clinically, when patients have been largely dosed with opium or its preparations. Over 130 experiments were made with apparatus for recording the blood pressure under the various methods of experimentation, and the tracings, reduced by means of photography, have been included in the report. By these tracings the case for the Commissioners is made much stronger, for anyone can see for himself the exactness of the observations, whatever conclusion he may draw from them.

It is impossible to state in detail the conclusions that the Commissioners came to, but the experiments, conducted, as they were, with the greatest possible care and avoidance of every conceivable impediment to correct observation, and hence incorrect deduction, leave no doubt in our mind that the Commissioners could come to no other conclusion than that which they obtained.

They concluded that "chloroform, when given continuously by any means which ensures its free dilution with air, causes a *gradual* fall in the mean blood pressure, provided the animal's respiration is not impeded in any way, and it continues to breathe quietly without struggling or involuntary holding of the breath . . . but if the chloroform is pushed further, there comes a time, not easy to define, when the blood pressure and respiration will no longer be restored spontaneously, although the heart continues to beat after the inhalation is stopped." Certain experiments were undertaken to observe the effect of stimulation and irritation of the vagus, and they tended to show that irritation—which after all is only over-stimulation—diminished the dangers of anæsthetics, for as the vagi became exhausted the blood-pressure would rise again. The slowing of the heart, following the irritation of the vagus, retards the absorption and conveyance of chloroform to the nerve centres, so that "of itself slowing or temporary stoppage of the heart in chloroform

14

administration is not dangerous." It is the violent respiratory efforts with bounding heart's action following any asphyxial state that constitutes the danger, for it leads to a rapid and dangerous inhalation of chloroform, and consequent rapid and dangerous fall of blood-pressure. And if "the chloroform is not removed from the face, one or both of two things will happen: (*a*) when the animal breathes again, it takes deep and gasping inspirations, the lungs become filled with chloroform, and an over-dose is taken in with extreme rapidity; or (*b*) when the restraining influence of the vagus is taken off the heart . . . the heart bounds on again, and the circulation is accelerated in proportion. The blood then becomes quickly saturated with chloroform, and an over-dose is at once conveyed to the nerve centres." We think this was a new theory on the action of the vagus in chloroform administration. The facts have been wrongly interpreted before.

The fourteen "practical conclusions" on page 75 form a most useful and succinct *résumé* of the deductions which may be drawn when viewed in a practical clinical light.

To complete the information on the dangers of anæthetics, the proprietors of the *Lancet* initiated a commission on the clinical aspect of the question, Dr. Dudley Buxton having undertaken to marshal the facts gathered. The materials for the report were obtained by sending round a series of questions to all hospitals in the United Kingdom with over ten beds, and to many on the Continent, in India, America, and the Colonies; and the anæsthetics enquired into were chloroform, ether, nitrous oxide, methylene, bromide of ethyl, ethidene dichloride, and various mixtures. An immense amount of information was collected, but the conclusion about the dangers of chloroform did not agree with that of the Commissioners of the second Hyderabad enquiry, for Dr. Buxton found that while chloroform was used more frequently, and the fatalities were proportionately greater from its use than from any other agent, in the majority of fatal cases the heart was stated to have failed before the respiration ceased. Dr. Buxton, however, brings forward the many well-known reasons which make all the difference between an experiment on an animal and the clinical observation on the human subject.

Cardiac failure during ether anæsthesia Dr. Buxton thinks due to the violence of the struggling, and not to the anæsthetic direct, and he expresses his belief, with which we heartily agree, that ether is the safest anæsthetic for general surgery.

This is only a brief outline of the general tenor of the report. Even if we do not accept the finding of the second Hyderabad Commission, the interpretation of their tracings, or the argument that what obtains in animals will probably do so in man, we cannot but admire the patience, perseverance, and skill of the observers, and their admirable and praiseworthy attempts to try and enlighten the many mysteries which obscure the real dangers attendant on the anæsthetic state. Nor must we forget a word of praise for the trouble the proprietors of the *Lancet* have taken in furnishing the medical profession with such a complete *résumé* of the controversy. Lastly, the thanks of the whole world are due to His Highness the Nizam of Hyderabad for his munificent support in the interests of science.

